# Cohesive Foil

**Published:** 1887-03

**Authors:** H. D. Boyd


### Cohesive Foil.

BY DR. H. D. BOYD.

(Read before the Alabama Dental Association.)

In place of a formal report on operative dentistry your committee offers you a paper believing that thereby the spirit of their work is better complied with than if a formal report with no paper were offered, and if in this paper there are faults it is yours to criticise; if old positions are taken instead of new, do not make the charge that originality is claimed for them.

The field is so broad, the work so important, that had it not been predetermined to elect one, and measurably leave other subjects to their fate, we would stand so appalled at our work that you perhaps would not be afflicted with even this. With one word more of appeal to your forbearance, or possibly sympathy, I will turn to the discussion of cohesive gold. That word is, that it is easier to think what you would like to say than to choose what you should say, particularly on a subject that has been so frequently and ably written on as this.

It was my lot but a few days ago to be called on by a lady of refinement and wealth who had been in the hands of a dentist of national repute. The teeth had been filled six times within the space of fifteen years, and more than one hundred dollars each time had been paid for filling, yet one filling alone remained in part out of six; the cavities were all in the superior incisors; these had lost substance by decay, by file, by fracture, until they stood as a tumble down ruin, wounding the pride and producing an ugly lisp of speech that made my fair patient lose much of the confident reliance on the skill and attainments of dentists. The right central will be used to illustrate, it being decayed on both lateral surfaces from below the gums and was broken away on the right obliquely from near the gums to the center of the incising surface, with no locking or anchorage room at this point; on the left the anterior or labial portion of the enamel stood so thin as to be transparent, while the palatine surface was gone somewhat in the shape of the half of the letter "u" inverted. This tooth was no worse than the other three. To fill this tooth the first thing done was to carefully remove soft decay, or pulpy matter, then with a chisel cut away jagged margins, then covering with the dam, a bit of waxed floss silk was tightly drawn around the neck of the tooth three times, bands and weights of course being used to keep the dam out of the way, then the more careful removal of decay was followed by cutting as broad and long a basal groove around the cervical wall as could be without too great a compromise of strength, then for the right side an angular but delicate groove was cut on both sides of the pulp to a point of union beyond the end of the nerve, near to the incising surface of the tooth, the utmost care being taken not to uncover the living nerve, and at the same time to leave as much of the bony structure of the tooth attached to the enamel as could be, then introducing a delicate covering of oxyphosphate cement as nonconductor, the gold was prepared by taking No. 4 cohesive foil, and with plier tearing it into square bits of assorted sizes, lifting a piece at a time with pliers in the right hand; with left forefinger and thumb it is rolled loosely into an egg-shaped pellet, long experience and careful experiment having taught me to take no stock in the teaching that the working qualities of gold are interfered with by handling with clean fingers. The finger-made pellet is used as being better adapted to this work than any machine-made pellet or cylinder.

Now, with clean pliers passing the gold through a spirit flame, it is carried at once to the cavity and placed first with a plugger by hand, then gently malleted into the basal groove most likely to easily retain it steadily in place, then adding pellet to pellet until the foundation is fully secured and the cervical margin of the cavity is well covered, then advancing along the grooves to the point of union, malleting each bit of gold well into place, having thus secured the locking flange into every possible place that will add strength to your filling, proceed to build out in the usual way so as to leave the tooth shaped as near like nature made it as the nature of the case will admit, using the mallet yourself (not an assistant), which, having both hands free, you can easily do.

Having spread this paper out to so much greater length than anticipated, I will go into no more details, but will say that to bring gold close—yes, so close to even the thinnest, most frail wall of enamel, so that it will not leake, and that too with the minimum risk of fracture—I find the mallet in my own hand a success, both as to labor saving and permanent results, having tried the various machine mallets, as well as well trained assistants; that all margins should be covered with gold, well beaten down when gold is the filling material used, that the more frail and delicate the tooth the greater the need to use gold if it be incisor or bicuspid, that in many cases it is far better to use alloys than tax yourself and patient with gold, if away from the front of the mouth, that it is easier, when once a familiarity with the material is acquired, to give proper protection to the tooth operated on, and reputation to yourself, by the use of the cohesive gold than non-cohesive; that while abundant space or access to the cavity should be had, wedging and the use of the chisel should be resorted to, the file being the poorest of all means for such and the most offensive to the patient.

